# Artificial intelligence-powered four-fold upscaling of human brain synthetic metabolite maps

**DOI:** 10.1177/03000605251330578

**Published:** 2025-04-21

**Authors:** Erin B Bjørkeli, Jonn T Geitung, Morteza Esmaeili

**Affiliations:** 1 Department of Diagnostic Imaging, 60483 Akershus University Hospital, Lørenskog, Norway; 2 Institue of Clinical Medicine, University of Oslo, Oslo, Norway; 3 Department of Electrical Engineering and Computer Science, 56627 University of Stavanger, Stavanger, Norway

**Keywords:** Artificial intelligence, deep learning, brain tumor, spatial resolution

## Abstract

**Objective:**

Compared with anatomical magnetic resonance imaging modalities, metabolite images from magnetic resonance spectroscopic imaging often suffer from low quality and detail due to their larger voxel sizes. Conventional interpolation techniques aim to enhance these low-resolution images; however, they frequently struggle with issues such as edge preservation, blurring, and input quality limitations. This study explores an artificial intelligence–driven approach to improve the quality of synthetically generated metabolite maps.

**Methods:**

Using an open-access database of 450 participants, we trained and tested a model on 350 participants, evaluating its performance against spline and nearest-neighbor interpolation methods. Metrics such as structural similarity index, peak signal-to-noise ratio, and learned perceptual image patch similarity were used for comparison.

**Results:**

Our model not only increased spatial resolution but also preserved critical image details, outperforming traditional interpolation methods in both image fidelity and edge preservation.

**Conclusions:**

This artificial intelligence–powered super-resolution technique could substantially enhance magnetic resonance spectroscopic imaging quality, aiding in more accurate neurological assessments.

## Introduction

Magnetic resonance spectroscopic imaging (MRSI) is a robust imaging modality for detecting tissue metabolites *in vivo*, but its clinical adoption remains limited. In brain examinations, MRSI provides valuable insights into neurological diseases such as gliomas.^1–3^ Despite its potential, the clinical application of MRSI is limited by several challenges, primarily including its long acquisition times and low spatial resolution.^4^ Achieving high-resolution MRSI, such as a matrix size of 128 × 128, is time-consuming and often unfeasible with current acquisition technologies.^4^ Consequently, most MRSI techniques are limited to lower resolutions, commonly 32 × 32 pixels (nominal voxel size of 7.5 cc for a field of view of 240 × 240 × 1 cm^3^), which hinders the detailed visualization and quantification of metabolites under clinical settings. A 32 × 32 grid setting is common in clinical brain MRSI, where a balance between resolution and acquisition time is needed.^4^

To overcome these challenges, interpolation methods have been explored to enhance the resolution of MRSI-derived metabolite maps.^5–7^ Traditional interpolation methods, such as nearest-neighbor and spline techniques, although commonly employed, often fail to preserve high-frequency details in metabolic maps, leading to blurred or inaccurate representations of metabolite distributions.^8^ Given these limitations, recent advancements in deep learning have introduced new horizons for improving image resolution and quality. Convolutional neural networks and other machine learning models have demonstrated success in image super-resolution tasks,^9,10^ making them a promising tool for enhancing metabolite maps derived from lower-resolution imaging data.

This study addresses the limitations of traditional interpolation techniques by introducing a deep learning–based approach to generate high-resolution metabolite maps, upscaling 32 × 32 images by four-fold. To train the deep learning model, we generated synthetic datasets derived from The Cancer Genome Atlas (TCGA) dataset,^11–13^ which includes magnetic resonance imaging (MRI) scans of gliomas (both lower-grade gliomas (LGGs) and glioblastomas). Using T1-weighted (T1w) and fluid-attenuated inversion recovery (FLAIR) MRI modalities, we generated higher-resolution 128 ×128 metabolite maps for three prominent metabolites—choline-containing metabolites (tCho), creatine (Cr) + phosphocreatine (PCr) given total creatine (tCr), and N-acetyl-aspartate (NAA)—as well as their corresponding metabolic ratios (tCho/NAA, tCho/tCr). These metabolites are examined as markers for tumor detection, characterization, treatment monitoring, and assessment of other neurological disorders.^1,2,14^

The primary objective of this study was to evaluate the potential of a deep learning model to upscale lower-resolution metabolite maps to higher-resolution ones. Moreover, we aimed to generate synthetic maps for gliomas by leveraging publicly available MRI datasets and improve visualization of metabolite distributions across brain regions, enhancing the overall diagnostic utility of MRSI. We compared the performance of our proposed deep-learning approach with two conventional interpolation methods—nearest-neighbor and spline interpolations.

## Materials and methods

We used MRI modalities such as T1w and FLAIR to generate synthetic higher-resolution metabolite maps. MRI anatomical images were sourced from the open-access TCGA dataset^11–13^ via The Cancer Imaging Archive repositories.^11,13,15^ TCGA datasets included MRI scans (including T1w and FLAIR) from both LGGs and glioblastomas (World Health Organization grade 4) (high-grade glioma (HGG)), the most aggressive malignancy. The BraTS20 challenge dataset included preprocessed MRI data from TCGA and associated tumor mask data of 450 participants. For this study, a dataset of 350 participants was used as a training dataset, and a dataset of 100 participants was used as a test dataset.

We selected 5–10 axial slices from each T1w and FLAIR image that contained tumor lesions with signal intensity greater than the mean intensity of the entire slice. Using these slices, we constructed 128 × 128 two-dimensional metabolic maps for three metabolites and two metabolic ratios, followed by construction of the corresponding 32 × 32 downsampled maps. Metabolite maps with matrix sizes of 128 × 128 and 32 × 32 correspond to voxel sizes of 1.718 × 1.718 × 10  and 6.875 ×6.875 × 10 mm³, respectively, assuming a slice thickness of 10 mm and a field of view of 220 × 220 mm. The metabolic maps focused on choline + glycerophosphocholine (tCho), Cr + tCr, NAA, tCho/NAA, and tCho/tCr. These metabolites were chosen as they are key markers for tumor detection and characterization as well as assessment of other disorders.^2^

**Figure 1.**
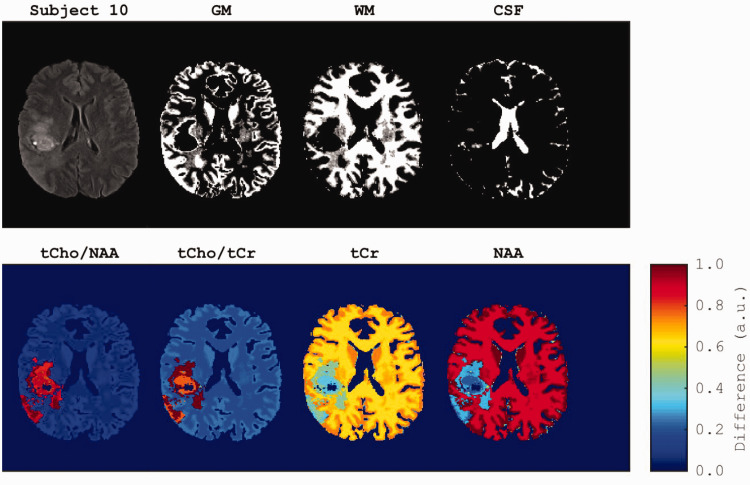
Example of an axial view of the fluid-attenuated inversion recovery image from participant 10, alongside the segmented regions (top row, from left to right): gray matter (GM), white matter (WM), and cerebrospinal fluid (CSF). Second row illustrates the corresponding metabolite ratios and two metabolites generated for this slice.

The BraTS dataset was preprocessed and skull-stripped. We performed segmentation of each brain to identify white matter (WM), gray matter (GM), and cerebrospinal fluid (CSF) using the FAST (FMRIB’s Automated Segmentation Tool) algorithm^16^ from the FSL software package,^17^ with default parameters. We then selected a few axial slices from each participant and resized them to a 128 × 128 pixel resolution (Figure 1).

To generate clinically relevant metabolite MRI data, the metabolite concentrations in GM and WM were determined based on their distinct metabolic profiles (Table 1). A metabolite map with 128 × 128 resolution was created by combining GM, WM, CSF (with zero concentration), and the tumor mask (with distinct metabolite concentrations), accounting for variations in metabolite concentrations across these regions. The formula used was as follows:

Higher−resolution metabolite maps=(Cratios or CGM)×GM+(Cratios or CWM)

 ×WM+CSF+(Cratios or estimated metabolite  levels  within  tumors) ×tumor masked region

**Table 1.** Metabolite concentration (range (mean)), based on the literature used for synthesizing metabolite maps.

	Concentration (mM)		
Metabolite	GM	WM	Tumor
tCr (Cr + PCr)	6.4–9.7 (8.05)	5.2–5.7 (5.45)	2.5–6.0 (4.25)
tCho (Cho + GPC)	1.6–2.0 (1.8)	1.3–1.6 (1.45)	2.5–5.5 (4.00)
NAA	8.0–11.0 (9.5)	6.0–9.0 (7.5)	1.5–3.5 (2.50)
	Tumor		
tCho/NAA	0.7–4.0 (2.35)		
tCho/tCr	0.5–3.0 (1.75)		
	Nontumor		
tCho/NAA	0.15–0.25 (0.20)	0.14–0.28 (0.22)	
tCho/tCr	0.40–1.40 (0.80)	0.25–1.20 (0.50)	

Cr: creatine; GPC: glycerophosphocholine; GM: gray matter; NAA: N-acetyl-aspartate; PCr: phosphocreatine; tCho: choline-containing metabolites; tCr: total creatine; WM: white matter.

The values in the parenthesis indicate the estimated concentrations in this study.

where C_GM_ and C_WM_ represent metabolite concentrations in normal adult brains, and C_ratios_ corresponds to estimated metabolite ratios in tumor lesions, as derived from previous studies^14,18–21^ (Table 1). To account for the differences between HGG and LGG in TCGA datasets, we made an assumption based on metabolite concentration ranges. Specifically, we used ratios below a certain threshold, defined as the average of the reported ranges, to construct a metabolite map of LGG lesions and employed ratios above the threshold to construct a metabolite map of HGG lesions. For nontumor lesions, we used metabolite ratio ranges above the average ratio for LGG. Subsequently, each metabolite map was normalized to ensure that pixel values ranged from zero to one.

To evaluate the model’s performance in real metabolite maps acquired by clinical MRSI, we used open-access data provided by Hingerl et al.^22^ The database consists of MRSI maps acquired from Vienna 7T scanner, with preprocessed metabolite maps in Medical Imaging Network Common data format. The files, with a matrix size of 64 × 64 × 39, were derived from two patients with glioblastoma.

### Network architecture

**Figure 2.**
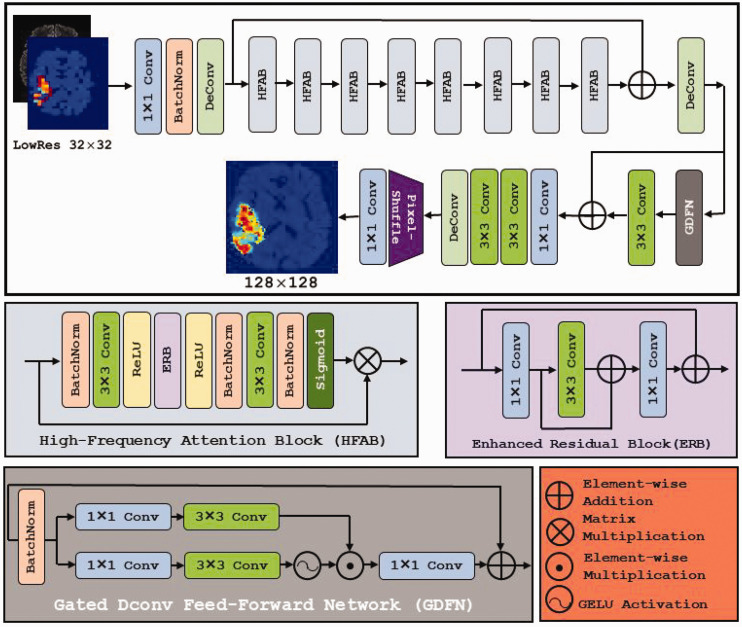
Deep learning model architecture for super-resolution metabolite maps includes specialized convolutional and attention-based blocks. Each block contributes to enhancing image resolution and preserving image details. The high-frequency attention block (HFAB) maintains sharpness and fine details, the enhanced residual block (ERB) preserves edges and textures, and the gated Dconv feed-forward network (GDFN) selectively filters important features.

Figure 2 provides an overview of our network architecture designed for super-resolution metabolite maps. The architecture incorporates feature extraction and reconstruction components that align with recent advancements in the field. Notably, a feature of our model is the high-frequency attention block (HFAB), which enhances high-frequency features critical for preserving fine details. Eight HFAB pairs were stacked sequentially, with two skipped connections to form the basis for the architecture of the network.

We incorporated a feed-forward network (FN) with a gating mechanism, using the gated Dconv feed-forward network (GDFN).^23^ The FN comprises two fully connected layers separated by a nonlinear activation, utilizing the element-wise product of two linear projection layers. This gating mechanism in the GDFN block regulates the passage of complementary features, improving image refinement and quality. Our approach to attention mechanisms, specifically the HFAB,^24^ introduces a sequential attention branch inspired by edge detection. This branch rescales each position based on its neighboring pixels, efficiently focusing on high-frequency areas. The HFAB employs a 3 × 3 convolution (3 × 3 conv, Figure 2) to expand the receptive field and improve computational efficiency.^24^ Batch normalization (BatchNorm) is integrated into the attention branch, incorporating global statistics during inference without additional computational cost. The HFAB block includes an enhanced residual block (ERB) that utilizes a 1 × 1 convolution to expand (the first element 1 × 1 conv, Figure 2) or contract (the last element 1 × 1 conv, Figure 2) the number of feature maps and a 3 × 3 convolution to extract features in a higher-dimensional space. Essentially, ERB allows efficient learning of high-dimensional features, which makes this block suitable for residual networks. Furthermore, two skip connections are integrated to mitigate training complexities. Through experimentation, we found that the model performed optimally with eight HFAB blocks, with no further improvements in loss, validation accuracy, image quality, or edge preservation beyond these levels. Fewer than eight HFABs led to suboptimal performance, suggesting that these HFABs provide the best balance between feature enhancement and efficiency. Additionally, the absence of the GDFN resulted in instability, poor validation accuracy, and limited generalizability. The inclusion of GDFN with its gating mechanism improved stability, refinement, and high-frequency feature preservation.

### Implementation details

The model was trained on the synthetic metabolite maps, with each map concatenated with its corresponding FLAIR image slice as a separate instance. The training dataset contained 15,000 metabolite maps (from 350 patients), of which 30% were used for validation during training. Each map included both a high-resolution version (target) and a downsampled low-resolution version, which served as the model’s input. Data augmentations, including random rotation and flipping, were applied to enhance model generalization. The model was developed using TensorFlow 2.5^25^ and Keras,^26^ with a total of 650,000 trainable parameters. We used the log hyperbolic cosine loss, which behaves similarly to L2 loss for small values and transitions to L1 loss for large values while remaining differentiable throughout.^27^ This loss function was minimized using the Adam optimizer.^28^ Training was conducted with a batch size of 24 and a learning rate of 1e^−5^, running for 100 iterations until convergence. The training was performed on a system with an NVIDIA A100 40 GB GPU and 94 GB of RAM, and approximately 3 GB of memory was used during training. After training, the model was evaluated on a not-before-seen separate test dataset of 100 patients, comprising 500 metabolite maps, to assess its performance.

### Evaluation metrics

Error maps were then created by subtracting the upscaled images from the corresponding ground truth high-resolution image generated for each participant in the test dataset. To assess the accuracy of reconstructed metabolite maps, we employed the mean squared error (MSE), as defined in Equation 1, and root mean squared error (RMSE), as defined in Equation 2, metrics to evaluate the error maps for each upscaling method. The statistical significance of performance differences between methods was assessed by comparing the mean differences of MSE and RMSE values using the Mann–Whitney *U* test, and the statistical significance threshold set at *p *< 0.05.

(1) $$\mathrm{MSE}\;=\;\frac{1}{N}\sum_{i=1}^{N}{\left(y_{i}-{\hat{y}}_{i}\right)}^{2}$$

(2) $$\mathrm{RMSE}\;=\sqrt{\frac{\sum_{i=1}^{N}{\left(y_{i}-{\hat{y}}_{i}\right)}^{2}}{N}}\;$$

Additionally, we used established metrics such as peak signal-to-noise ratio (PSNR), as defined in Equation 3, structural similarity index (SSIM),^14^ as defined in Equation 4, and learned perceptual image patch similarity (LPIPS).^29^ The LPIPS measures the high-level similarity between images and thus correlates well with human perceptual judgment. The PSNR, SSIM, and LPIPS metrics were compared between each pair of methods using the Mann–Whitney *U* test.

(3) $$\mathrm{PSNR}=20.\log_{10}\left({\mathrm{MAX}}_{I}\right)-10.\log_{10}(\mathrm{MSE})$$

where MAX_I_ indicates the maximum pixel value of the image.

(4) $$\mathrm{SSIM}=\frac{\left(2\mu_{x}\mu_{y}+c_{1}\right)(2\sigma_{xy}+c_{2})}{\left(\mu_{x}^{2}+\mu_{y}^{2}+c_{1}\right)(\sigma_{x}^{2}+\sigma_{y}^{2}+c_{2})}$$

where μ_*x*_ and μ_*y*_ represent the pixel sample mean of x and y, respectively; $\sigma_{x}^{2}\;{\mathrm{and}\;\sigma }_{y}^{2}$ indicate the sample variance of x and y, respectively; σ_*xy*_ indicates the sample covariance of x and y; c_1_ = (k_1_L)^2^ and c_2_ = (k_2_L)^2^ are two variables to stabilize the division with a weak denominator; L indicates the dynamic range of the pixel values (typically 2^#bits per pixel^−1); k_1_ = 0.01; and k_2_ = 0.03.

## Results and discussion

**Figure 3.**
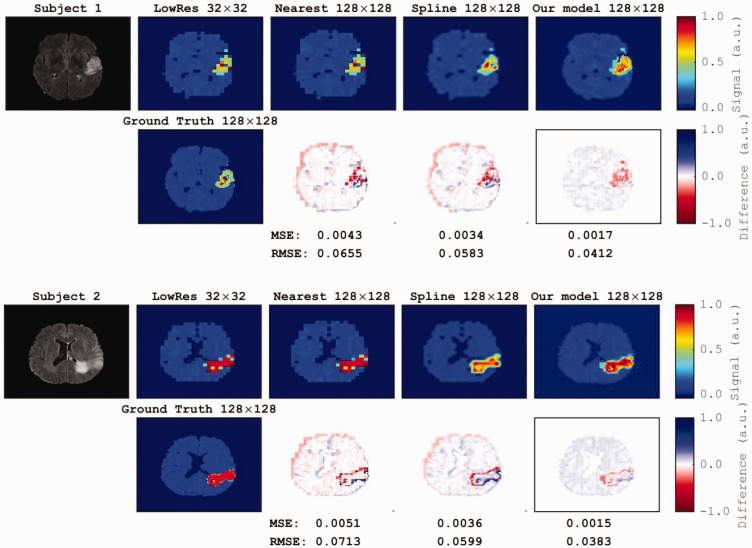
A comparison of our model’s upscaling performance with two common interpolation methods is shown in two examples from the test dataset. For each participant, the second row illustrates the ground truth high-resolution image, followed by the differences generated by subtracting the ground truth high-resolution (HighRes 128 × 128) image from each upscaled image in the first row. Corresponding MSE and RMSE measurements for the differences between each upscaled slice and the ground truth are provided for each upscaling method. MSE: mean squared error; RMSE: root mean squared error.

**Figure 4.**
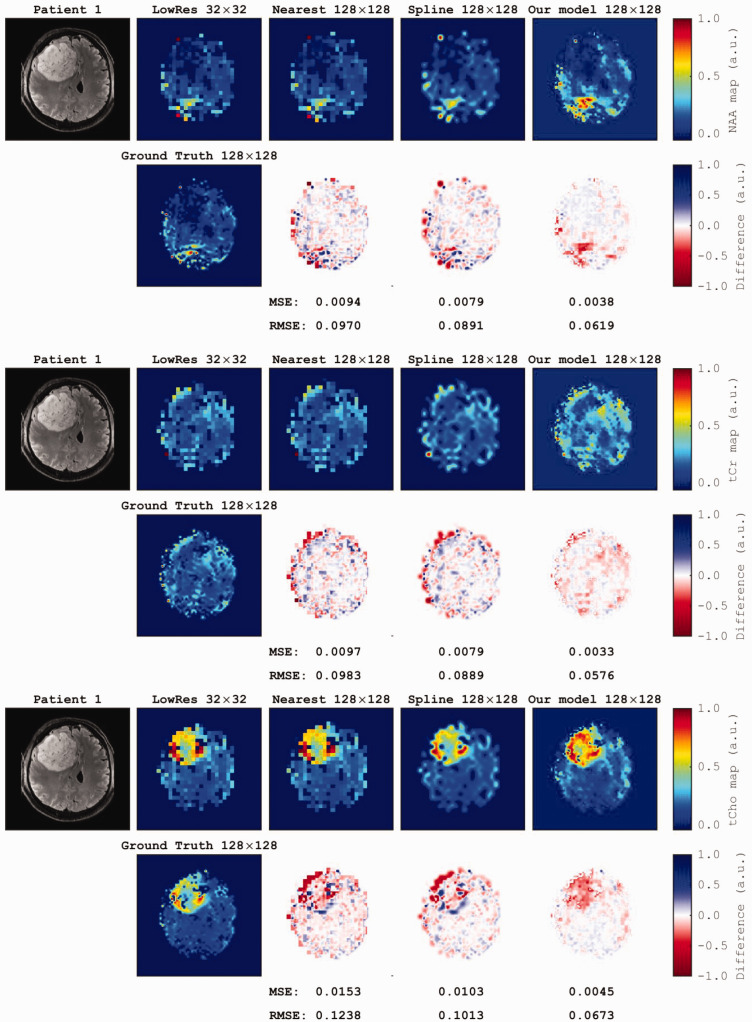
Axial section of MP2RAGE and metabolic maps obtained using a 7T magnetic resonance imaging system and 3D-CRT-MRSI (64 × 64 × 39 matrix) for a patient with glioma (male, 51 years, newly diagnosed glioblastoma, WHO grade IV, frontal left). Metabolite maps for NAA, tCr, and tCho showing reduced levels of NAA and tCr in the necrotic region, whereas tCho level was increased in viable tumor tissue. Additionally, the second row of each section shows the ground truth high-resolution images, whereas the Continued.first row presents the differences generated by subtracting the ground truth from each upscaled image using the DL model and two common interpolation methods. The corresponding MSE and RMSE measurements are provided for each upscaling method. MP2RAGE: magnetization-prepared 2 rapid gradient echoes; 3D-CRT-MRSI: three-dimensional concentric ring trajectory magnetic resonance spectroscopic imaging; WHO: World Health Organization; NAA: N-acetyl aspartate; tCr: total creatine; tCho: total choline.

Low-resolution 32 × 32 metabolic maps were upscaled four-fold using our model and compared with nearest-neighbor and spline interpolation techniques (Figure 3). We began with a low resolution typically used for MRSI, aiming for a reasonable scan time. However, when starting with a lower resolution of 16 × 16, the images lacked sufficient detail to produce satisfactory results, which is consistent with the findings from previous studies (e.g. Iqbal et al.^9^ and Dong et al.^7^). The differences between the ground truth and upscaled images were computed via pixel-wise subtraction. Nearest-neighbor and spline techniques resulted in interpolated images at a resolution of 128 × 128 pixels, with mean MSEs of 0.0048 ± 0.0020 and 0.0037 ± 0.0012, respectively, compared with the ground truth images, whereas the mean RMSEs were 0.0706 ± 0.0183 and 0.0599 ±0.0826, respectively. The discrepancies between the upscaled maps and ground truth maps reached a significant threshold when comparing the mean MSEs between our model (0.0018 ± 0.0008) and nearest-neighbor (Mann−Whitney *U* test, *p* < 0.0001) and spline (*p *= 0.006) methods. The error maps revealed a significant difference in RMSEs between our model (0.0406 ± 0.0142) and the nearest-neighbor (*p *= 0.002) and spline (*p *=* *0.034) methods. The error maps derived from the clinical MRSI dataset of two patients showed a significant decrease in MSE (0.0016 ± 0.0030) and RMSE (0.0396 ± 0.0187) of metabolite maps derived from our model compared with the nearest-neighbor (MSE =0.0044 ± 0.0025, *p *= 0.008; RMSE =0.0691 ± 0.0203, *p *= 0.006) and spline (MSE = 0.0035 ± 0.0012, *p *= 0.007; RMSE = 0.0591 ± 0.0143, *p *= 0.004) methods (Figure 4).

Quantitative evaluation based on PSNR, SSIM, and LPIPS consistently demonstrated that our model provided higher-quality upscaling compared with the nearest-neighbor and spline techniques. Our model achieved a PSNR of 29.57 dB, which was significantly higher than those of nearest-neighbor (21.87 dB, *p *< 0.0001) and spline (23.28 dB, *p *< 0.0001) interpolations (Table 2). This indicates that our model preserves a large amount of the original image information during the upscaling process. The SSIM score of our model was 0.92, which was significantly higher than those of nearest-neighbor (0.76, *p *< 0.0001) and spline (0.77, *p *< 0.0001) interpolations. This suggests that our model maintains a higher degree of structural similarity to the ground truth image, preserving key details such as metabolite boundaries and regional contrasts. Regarding LPIPS, our model achieved a score of 0.08, demonstrating a closer resemblance to the ground truth high-resolution image, and was significantly lower than the scores of nearest-neighbor (0.26, *p *< 0.0001) and spline (0.17, *p *< 0.0001) interpolations. This indicates that our model provides a more visually realistic reconstruction of the metabolite maps.

**Table 2.** Evaluations of different upscaling methods using metrics of structural similarity index (SSIM), peak signal-to-noise ratio (PSNR), and learned perceptual image patch similarity (LPIPS).

	PSNR	SSIM	LPIPS
Our model	29.5745 (3.7339)	0.9196 (0.0478)	0.0856 (0.0423)
Spline	23.2894 (3.8814)	0.7718 (0.0731)	0.3810 (0.0575)***
Nearest	21.8766 (3.9699)	0.7686 (0.658)	0.2609 (0.0592)***

Results are presented as mean (standard deviation). Statistically significant differences are shown with***.

Despite the utility of image fidelity assessment based on SSIM, PSNR, and LPIPS in model evaluations, these metrics may not fully capture the clinical relevance of the reconstructed metabolite maps (e.g. in the presence of MR image distortion).^30^ These metrics focus on technical aspects such as structural similarity and perceptual appearance, which are useful for comparing the performance of our model with other techniques. However, they do not reflect how well the maps support clinical decision-making. To improve clinical relevance, we included FLAIR images as input to our model. This helps preserve the biological structure and anatomical context, enhancing the accuracy of the metabolite maps. We acknowledge that future work should involve clinical validation using metrics tied to real-world applications, such as expert evaluations, to better assess the practical value of the reconstructed maps.

A previous study used the multi-scale UNet architecture,^7^ which provided an average PSNR of 30.86 dB, an SSIM score of 0.9477, and an LPIPS score of 0.03 when upscaling metabolite maps to 64 × 64, evaluated on a dataset of 15 patients. In their study, due to the limited number of patients, they reported the results as average performance on five-fold cross-validation. Another similar study used the densely connected UNet,^9^ which resulted in an MSE of <0.008 between the ground truth and metabolite maps upscaled to 128 × 128. Despite the similarity in quantitative results between our study and these recently published studies, we could not directly compare our model’s performance with that of their approach, acknowledging that such a comparison may not be entirely reasonable at this stage. The models in these studies were trained on different datasets, and the network structures may not be identical, which could introduce confounding factors. Given that our focus was on comparing traditional interpolation methods, future work will aim to extend these comparisons by incorporating more advanced deep learning–based models once appropriate datasets and resources become available.

Overall, the error maps and quantitative metrics suggest that our deep learning–based model outperforms traditional interpolation techniques, providing higher-resolution metabolite maps with relatively higher image quality compared with nearest-neighbor and spline interpolations. This demonstrates its superior performance in both objective image and perceptual similarity. Higher-resolution metabolite maps can enhance the visualization of prominent metabolites, such as tCho, tCr, and NAA, as well as their ratios, which provide complementary diagnostic values for tumor detection, characterization, and treatment monitoring in gliomas.^6^

MRSI has long been recognized as a valuable and promising *in vivo* biomedical imaging modality that enables the detection of brain metabolites.^2,14^ However, despite its potential, limitations such as spatial resolution hinder its widespread use in clinical applications. Acquiring MRSI with an in-plan higher resolution, such as a 128 × 128 resolution using 3T MRI systems, is prohibitively time-consuming and nearly impossible with existing hardware and acquisition technologies. Thus, proper postprocessing including interpolation becomes a crucial step in enhancing the visualization of metabolite maps derived from MRSI modalities.^4^ In this study, we addressed the limitations of conventional interpolation methods, proposing the potential application of a deep-learning approach to improve the visualization of metabolite maps.

The limited availability of MRSI-derived metabolite maps has posed a significant challenge for researchers in the spectroscopic community, restricting progress in the field. To address this, we proposed an alternative approach that generates metabolite maps using publicly available datasets. By incorporating knowledge from existing studies on metabolite concentrations in the GM and WM of healthy adult brains, along with information on metabolites and their ratios in tumor lesions, we could create a dataset for training and testing our model. Although our results show great promise, several important limitations should be acknowledged. The synthetic metabolite maps were generated based on established concentrations in normal brain tissue and glioma lesions, as reported in the literature. Although these synthetic data closely resemble real MRSI acquisitions, they are based on certain assumptions and do not capture the full complexity of real-world data. For instance, our model does not account for acquisition artifacts, such as motion, partial volume effects, or magnetic field inhomogeneities, which can significantly impact the quality of actual MRSI data. Additionally, variations in acquisition protocols, imaging systems, and patient populations in clinical environments can introduce further complexities not reflected in the synthetic data used here. Consequently, although our method shows promise, further validation using real-world clinical data is necessary to assess its robustness and generalizability in diverse clinical scenarios. To improve the model’s applicability, future studies should incorporate these covariates, so that the metabolite maps have more refined specifications that reflect real-world challenges. Additionally, testing the model on datasets with varying levels of noise, resolution, and disease severity would be crucial to assess its robustness and generalizability across a wider range of clinical conditions. Furthermore, future work would benefit from clinical validation, such as evaluations by radiologists or use of clinically relevant metrics, to better assess the practical value of the reconstructed metabolite maps under clinical settings.

Regarding the ethical and practical implications of deploying this technology in clinical settings, we acknowledge that our model is not yet ready for clinical deployment at this stage. This study primarily focused on exploring recent advancements in super-resolution (SR) models within the context of MRSI. Notably, MRSI operates at a lower resolution than typical low-resolution imaging in SR challenges, with in-plane spatial resolutions of 64 × 64 pixels or lower, which is often subject to eight-fold upscaling. This raises the question of whether the model structures we developed can be effectively applied in clinical cases in which low resolution is a persistent and significant challenge.

In summary, this study highlights the potential of deep learning to enhance the resolution and quality of the lower-resolution metabolite maps generated. Our model outperformed traditional interpolation methods in image quality and perceptual similarity, offering a promising path to improving MRSI’s clinical utility. However, further refinement with real-world data and more complex acquisitions are needed for broader clinical application, along with addressing limitations in synthetic data, acquisition parameters, and whole-brain coverage. This study marks a step forward in making high-resolution MRSI more accessible and relevant for brain tumor imaging and neurological disorders. In future work, we aim to conduct studies involving clinical data to evaluate how the enhanced metabolite maps impact tumor detection and monitoring, providing a clearer understanding of their role in clinical decision-making.

## Data Availability

The data that support the findings of this study are available from the corresponding author. The code is available at https://github.com/MorEsm/SR-MRSI.
